# The Effect of ‘Integrated Health Promotion Initiative’ on Awareness among Opinion Leaders Regarding Hypertension

**DOI:** 10.4103/0970-0218.39251

**Published:** 2008-01

**Authors:** AR Dongre, PR Deshmukh, BS Garg

**Affiliations:** Department of Community Medicine, Mahatma Gandhi Institute of Medical Sciences, Sewagram, Maharashatra, India

## Introduction

The National Cardiovascular Diseases (CVDs) Control Program in India is based on the strategies of population approach of primordial and primary prevention.(1) It requires community mobilization to alter its life-style practices associated with non-communicable diseases (NCDs) like sedentary habits, excess diet, obesity, alcohol consumption, and tobacco and salt intake.(1) The Department of Community Medicine, Mahatma Gandhi Institute of Medical Sciences (MGIMS), Sewagram, implemented Integrated Health Promotion Initiatives (IHPI) program in two primary health centres of Wardha district. The aim of IHPI program was to assess the current status of health promotion initiatives in the community and to build capacity for health promotion at various levels. The present study reports the outcomes of the program efforts on the level of awareness regarding symptoms, causation and availability of treatment facility for hypertension among opinion leaders from program area. Here, hypertension was viewed as an ‘Index Disease’ as it shares risk factors common with other NCDs, is easy to detect and diagnose in field settings, and is easily accepted by the patients and the community (no stigma and less apprehension).

## Materials and Methods

During January-February 2003, 272 opinion leaders from all 43 villages of Anji and Gaul Primary Health Centre area of Wardha district were interviewed by medical personnel using pre-designed, pre-tested, semi-close-ended questionnaire. Opinion leaders were village panchayat members, leaders of women's self-help groups (SHGs), religious leaders, teachers and other prominent persons in the village. The responses were recorded on multiple response sheets without any prompt. For all those who could not be contacted in the first instance, two further visits were made before declaring the subject unavailable. It was assumed that these opinion leaders were more likely to be consulted by villagers for seeking treatment.

Over the period of two years, a trained team, comprising of a doctor, a medical intern, ANM and a social worker, organized health awareness campaigns and conducted diagnostic camps for NCDs at each village. Referral, if required, was given to the nearest available health facility. During the program period, monthly orientation programs focusing on NCDs and environmental sanitation were held for opinion leaders, PHC staff, *anganwadi* workers and representatives of local partner NGOs. Later, in February-March 2005, 250 opinion leaders were interviewed again using the same questionnaire to study the effect of program on their level of awareness.

## Results

In 2003 and 2005, 272 and 250 opinion leaders were interviewed, respectively [Table 1]. There was improvement in their knowledge regarding symptoms of hypertension. Awareness of symptoms like palpitation and breathlessness significantly increased from 30.9% to 45.2% and 23.5% to 42.8%, respectively [Table 2].

**Table 1:** Distribution of opinion leaders who participated in the study

Opinion leader	Year 2003 (n = 272)	Year 2005 (n = 250)
Sarpanch	41 (15.1)	34 (13.6)
Other panchayat members	50 (18.4)	37 (14.8)
Self help group leaders	64 (23.5)	70 (28.0)
Teachers	68 (25.0)	60 (24.0)
Religious leaders	19 (7.0)	25 (10.0)
Other informal leaders	30 (11.0)	24 (9.6)

Figures in parenthesis are percentages

**Table 2:** Knowledge of opinion leaders regarding symptoms, risk factors and facility to seek treatment for hypertension in year 2003 and year 2005

	Year 2003 (n = 272)	Year 2005 (n = 250)
Knowledge regarding symptoms of hypertension		
Headache	60 (22.5)	94 (37.5)
Giddiness	96 (35.3)	99 (39.6)
Palpitation*	84 (30.9)	113 (45.2)
Breathlessness*	64 (23.5)	107 (42.8)
Blurring of vision	28 (10.3)	23 (9.2)
Knowledge regarding risk factors of hypertension		
Lack of exercises	40 (14.7)	68 (27.2)
Excess diet	76 (27.9)	104 (41.6)
Tobacco/alcohol	36 (13.2)	14 (5.6)
Mental stress*	76 (27.9)	134 (53.6)
Hereditary	12 (4.4)	7 (2.8)
Person/facility to seek treatment for hypertension		
No need of treatment	8 (2.9)	1 (0.4)
Primary health centre*	52 (19.1)	94 (37.6)
Private doctor	20 (7.4)	61 (24.4)
District/medical college hospital*	148 (54.4)	102 (40.8)
Ayurvedic/homeopathic doctor	4 (1.5)	0 (0)

Figures in parentheses are percentages,

* *P* < 0.05

There was improvement in awareness regarding excess diet, lack of exercise as causes of hypertension. There was significant improvement in awareness regarding mental stress as cause of hypertension from 27.9% to 53.6% [Table 2] and a significant positive shift in respondents' opinion from 19% to 37.6% for primary health centre as the source of treatment [Table 2].

The program could also improve treatment seeking and their compliance. The proportion of known hypertensive cases increased significantly from 13.6% to 37.9% during these two years of intervention (*P* < 0.05). The proportion of patients who took treatment regularly also increased from 8.7% to 12.3% and those practicing meditation regularly also increased from 0.7% to 1.9%.

## Discussion

Although there was improvement in specific knowledge on hypertension among respondents, which was poor in the beginning [Table 2], none of the respondents could tell that hypertension rarely shows any symptom. It could be because the awareness regarding symptoms of individual NCD was difficult to achieve in integrated approach of short duration. A long-term nationwide cardiovascular disease control program in a developing country like Seychelles could make 28% of the adult participants (25-64 years) know that hypertension rarely shows symptoms.(2) As seen in Table 2, the overall knowledge regarding various risk factors of hypertension shows improvement, which was difficult to achieve. The ‘CVD risk management package’ of WHO emphasizes a shift of focus from the treatment of individual risk factor to comprehensive cardiovascular risk management.(3)

In the present study, a significant number of respondents reversed their opinion from district/medical college hospital and identified primary health center as the source of treatment for hypertension [Table 2], as hypertension can be detected and managed easily at primary health centre. Community's belief that treatment is only available at district hospital may serve as hindrance in accessing treatment and their compliance. The awareness of people and availability of services at the present primary health care system needs to be strengthened for management and prevention of hypertension.

## Conclusion

The Integrated Health Promotion Initiative program in two primary health centres of Wardha district could improve the awareness regarding symptoms, risk factors and availability of treatment among the opinion leaders to whom the villagers usually go for consultation. However, more intensive and sustained efforts are required for significant impact. Hence, there is a need to up-scale and sustain integrated health promotion initiatives.
